# Definition of a novel breast tumor-specific classifier based on secretome analysis

**DOI:** 10.1186/s13058-022-01590-4

**Published:** 2022-12-20

**Authors:** Philémon Sirven, Lilith Faucheux, Maximilien Grandclaudon, Paula Michea, Anne Vincent-Salomon, Fatima Mechta-Grigoriou, Alix Scholer-Dahirel, Maude Guillot-Delost, Vassili Soumelis

**Affiliations:** 1 INSERM Unit U932, Immunity and Cancer, Institut Curie, Paris, France; 2 Paris Sciences Lettres (PSL) University, Paris, France; 3 Center of Clinical Investigation, CIC IGR-Curie 1428, Paris, France; 4 INSERM U976, Université de Paris, IRSLHôpital Saint Louis, 75006 Paris, France; 5 INSERM UMR1153, Université de Paris, ECSTRRA Team, 75006 Paris, France; 6 Diagnostic and Theranostic Medicine Division, Institut Curie, Paris, France; 7 Centre de Recherche, Stress and Cancer Laboratory, U830 Genetics and Biology of Cancers, INSERM, Institut Curie, Paris, France; 8 Department of Immunology-Histocompatibility, AP-HP, Hôpital Saint-Louis, 75010 Paris, France

**Keywords:** Breast cancer, Secretome, Tumor, Juxta-tumor, Breast tumor-specific signature

## Abstract

**Background:**

During cancer development, the normal tissue microenvironment is shaped by tumorigenic events. Inflammatory mediators and immune cells play a key role during this process. However, which molecular features most specifically characterize the malignant tissue remains poorly explored.

**Methods:**

Within our institutional tumor microenvironment global analysis (T-MEGA) program, we set a prospective cohort of 422 untreated breast cancer patients. We established a dedicated pipeline to generate supernatants from tumor and juxta-tumor tissue explants and quantify 55 soluble molecules using Luminex or MSD. Those analytes belonged to five molecular families: chemokines, cytokines, growth factors, metalloproteinases, and adipokines.

**Results:**

When looking at tissue specificity, our dataset revealed some breast tumor-specific characteristics, as IL-16, as well as some juxta-tumor-specific secreted molecules, as IL-33. Unsupervised clustering analysis identified groups of molecules that were specific to the breast tumor tissue and displayed a similar secretion behavior. We identified a tumor-specific cluster composed of nine molecules that were secreted fourteen times more in the tumor supernatants than the corresponding juxta-tumor supernatants. This cluster contained, among others, CCL17, CCL22, and CXCL9 and TGF-β1, 2, and 3. The systematic comparison of tumor and juxta-tumor secretome data allowed us to mathematically formalize a novel breast cancer signature composed of 14 molecules that segregated tumors from juxta-tumors, with a sensitivity of 96.8% and a specificity of 96%.

**Conclusions:**

Our study provides the first breast tumor-specific classifier computed on breast tissue-derived secretome data. Moreover, our T-MEGA cohort dataset is a freely accessible resource to the biomedical community to help advancing scientific knowledge on breast cancer.

**Supplementary Information:**

The online version contains supplementary material available at 10.1186/s13058-022-01590-4.

## Introduction

Inflammation is a naturally selected process that has evolved to protect healthy tissues from damage. Pathological processes such as cancer occur in the context of specific inflammatory situations [1, 2]. During tumorigenesis, the healthy tissues adapt to the global changes occurring at the microenvironment level in the attempt of maintaining tissue integrity and homeostasis. Hence, the systematic study of healthy tissue alongside its tumor counterpart is paramount to understanding those modifications [3–6]. Specific inflammatory features that arise and develop within the normal tissue microenvironment should be different and specific to each tissue type, thus to each cancer type.

Inter- and intra-tissue diversity reflects cancer heterogeneity, which is representative of different molecular, pathological, and clinical tumor subgroups. Patient cohorts should be large enough when setting up tumor biology studies in a way that fundamental observations and new findings are of clinical relevance.

Alongside the importance of studying normal tissue in comparison with tumor samples and the need for large patients’ cohorts, the methodology and technology used to investigate the complexity of the tumor microenvironment are essential. Currently, the majority of studies are focusing on the detailed analysis of the tumor tissue, either from the tumor cell point of view or from the immunological perspective*.* Many techniques have been developed and applied to tackle cancer complexity, including single-cell resolution methods such as flow cytometry, mass cytometry [5, 7, 8], and more recently single-cell RNAseq. Bulk tumor transcriptomics approaches [9–11] remain of interest for studying tumor composition. The Cancer Genome Atlas Program is today a reference in terms of genomic characterization of different types of cancer and matched normal tissue. However, we hypothesized that genomic data may only partially reflect specific tumor inflammatory elements, such as cell composition and soluble mediators that can be transiently secreted or produced at particularly low levels.

Studies at the protein level are necessary to precisely define specific features of cancer inflammation. This requires a systematic comparative approach of the tumor tissue with the non-tumor counterpart, in large patient cohorts. In the attempt to comprehensively understand cancer complexity, integrating data from different sources is of primary importance because it allows to mathematically reduce this complexity and formalize specific cancer signatures. In order to do so, we decided to perform a medium-scale analysis of tissue-derived secretome from 422 primary human breast cancer (BC) patients, as well as paired corresponding non-involved juxta-tumor breast tissue. We provide a novel and highly predictive classifier defining breast tumor inflammation.

## Material and methods

### Human samples and patients’ characteristics

Fresh samples of tumoral and juxta-tumoral (adjacent to the tumor and exempt of malignant tumor cells) tissues of 422 untreated breast cancer (BC) patients were obtained within three hours after surgical resection from the Department of Pathology (Institut Curie hospital, Paris) as surgical residues. The surgical specimens were received fresh from the operative room within less than 30 min. Tumoral and juxta-tumoral tissues were selected during the macroscopic examination of the surgical specimens by a pathologist. The juxta-tumoral tissues were assessed as being normal breast parenchymas by specialized breast pathologists at the macroscopic examination as well as using frozen tissue sections. No juxta-tumor subgroups were found according to the surgery type (i.e., mastectomy, tumorectomy) or molecular breast cancer subtypes (Table S6, data not shown). The Internal Review Board and Clinical Research Committee of the Institut Curie approved this study, named T-MEGA (tumor microenvironment global analysis), and samples were included prospectively between 2011 and 2016. All patients gave informed consent for research use of their biologic material in accordance with the declaration of Helsinki. The T-MEGA study was conducted in a laboratory that operates under exploratory research principles. Samples were collected based on the following inclusion criteria: age > 18 years, pathological diagnosis of breast cancer, untreated tumors, absence of immune-modulating factors (including steroids) within the past month, and absence of neoadjuvant therapy. Patient characteristics are summarized in Table 1.

**Table 1:** Breast cancer patient cohort characteristics

	Features levels	Secretome (n = 422)	Glucose–Lactate (n = 38)
Age at diagnosis	*med [range]*	59.2 [22.4–92.9]	60.8 [22.4–88.8]
BMI	*med [range]*	24.3 [15.7–45.5]	25 [16.6–39.2]
Pregnancy: *n (%)*	No	61 (14.8%)	6 (16.2%)
	Yes	351 (85.2%)	31 (83.8%)
	NA	1	1
Menopause: *n (%)*	No	109 (29.2%)	7 (20%)
	Yes	264 (70.8%)	28 (80%)
	NA	3	2
Age at first relapse event	*med [range]*	64 [32.3–94.8]	63.4 [38–82.9]
pT: *n (%)*	pT1a	5 (1.2%)	2 (5.3%)
	pT1b	8 (1.9%)	0
	pT1c	180 (42.7%)	14 (36.8%)
	pT2	208 (49.3%)	21 (55.3%)
	pT3	20 (4.7%)	1 (2.6%)
	pT4b	1 (0.2%)	0
pN: *n (%)*	pN0	208 (49.3%)	20 (52.6%)
	pN1	146 (34.6%)	11 (28.9%)
	pN2	44 (10.4%)	3 (7.9%)
	pN3	24 (5.7%)	4 (10.5%)
EE grade: *n (%)*	1	25 (6%)	1 (2.6%)
	2	182 (43.3%)	13 (34.2%)
	3	213 (50.7%)	24 (63.2%)
	NA	2	0
Ki-67%: *n (%)*	< 20	140 (36.4%)	9 (42.9%)
	≥ 20	245 (63.6%)	12 (57.1%)
	NA	3	2
Lesional code: *n (%)*	Ductal (INV)	331 (78.4%)	33 (86.8%)
	Ductal and Lobular (INV)	8 (1.9%)	1 (2.6%)
	Lobular (INV)	50 (11.8%)	3 (7.9%)
	Other (INV)	33 (7.8%)	1 (2.6%)
Vascular emboli: *n (%)*	No	236 (56.2%)	15 (39.5%)
	Yes	184 (43.8%)	23 (60.5%)
	NA	2	0
Molecular class (ER, HER2): *n (%)*	HER2 (±)	28 (6.6%)	8 (21.1%)
	LUMA (±, ki-67 < 20)	130 (30.8%)	6 (15.8%)
	LUMB (±, ki-67 ≥ 20)	158 (37.4%)	6 (15.8%)
	LUMHER2 (+ +)	30 (7.1%)	8 (21.1%)
	TN (–)	76 (18%)	10 (26.3%)

Number of samples and percentage per level of the categorical clinical variables, and median with range (minimum–maximum) for continuous variables are shown for each dataset

### Tissue supernatants production

All the tissues were transported in a CO2-independent medium (Gibco) and processed within three hours after surgical resection. Breast tumor and juxta-tumor tissues were cut in pieces of 15-20 mg. Each piece of tissue was put in one well of a 48-well plate in 250 µL of culture medium (RPMI GlutaMAX (Gibco)) containing 10% FBS (HyClone), 1% sodium pyruvate, 1% non-essential amino acids 100X, 1% penicillin/streptomycin (Gibco) without any stimulation. Supernatants were harvested after 24 h at 37 °C. Supernatants were diluted 1:2 (v/v) with culture medium (description above), filtered with a 0.22 µm filter (Millipore), stored at -80 °C, and then used to quantify the secretome.

### Tissue secretome

Concentrations of 51 different molecules were measured in tissue supernatants using five bioplex kits assays. Human Milliplex Map kits (Human MMP magnetic Bead panel 2, Human Adipocyte Magnetic Bead Panel and Human cytokine/chemokine Magnetic Bead panels I, II, III) were purchased from Millipore (#HMMP2MAG, HADCYMAG, #HCYTOMAG, #HCYP2MAG, #HCYP3MAG) and used according to manufacturer’s recommendations. The following molecules were simultaneously measured: Adiponectin, CCL1, CCL2, CCL3, CCL4, CCL5, CCL7, CCL8, CCL17, CCL20, CCL22, CXCL5, CXCL6, CXCL7, CXCL8, CXCL9, CXCL10, CXCL12 (a + b), EGF, FGF-2, G-CSF, GM-CSF, GRO, HGF, IL-1β, IL-1RA, IL-6, IL-9, IL-12p40, IL-12p70, IL-15, IL-16, IL-21, IL-23, IL-33, Leptin, LIF, M-CSF, MMP1, MMP2, MMP9, Resistin, SCF, Serpin E1, TGF-α, TNF-α, TNF-β, TPO, TRAIL, TSLP, and VEGF. Data were acquired using a BIO-PLEX 200 plate reader and analyzed with the Bio-Plex Manager 6.1 software. IL-10 and TGF-β1, TGF-β2, TGF-β3 were measured with electro-chemiluminescent detection method (MSD). The V-PLEX Proinflammatory Panel 1 kit (K151QUD) and the U-PLEX TGF-β Combo human kit (K15241K) were purchased from MSD and used according to the manufacturer’s instructions. Data were acquired using a MESO QUICKPLEX SQ 120 plate reader and analyzed with the DISCOVERY WORKBENCH 4.0 software. For each analyte, concentrations below the lower limit of detection of the corresponding batch were imputed by a common value. This value was set as half of the mean lower detection limits over the batches that included out-of-range samples. Similarly, for each analyte, concentrations above the upper limit of detection of the corresponding batch were imputed by the maximum of the upper detection limits of the analyte. These lower and upper detection limits are reported in Table S2. Some concentrations were missing for some samples because of beads aggregation, which led to an insufficient number of acquired beads.

### Colorimetric assay

Glucose and lactate concentrations were measured from supernatants using a colorimetric assay. The Glucose Assay Kit and the Lactate Assay Kit were purchased from Abnova (KA0831 and KA0833) and used according to the manufacturer’s recommendations. To fit into the linear concentration range of the assay, samples were diluted at 1:200 for glucose assay and 1:50 for lactate assay. Data were acquired using a Fluostar OPTIMA BMG Labtech plate reader and analyzed with the optima data analysis software (version 3.32).

### Statistical analysis

#### Low detection

IL-9, TPO, IL-12p40, TNF-β, TSLP, and IL-21 concentrations were detected in less than 5% of the normal tissue samples (juxta-tumors) and were therefore excluded from multiparametric analyses, except for the secretome-based distance.

#### Data preprocessing

The secretome concentration, glucose consumption, and lactate production exhibited log-normal distributions. A logarithmic (base 10) transformation was performed for all these variables, including ratios, for visual representation and before each analysis: tests, correlations, linear and elastic-net regressions, principal component analysis (PCA), and clustering.

#### Secretome-based distance

A secretome-based distance between paired tumor and juxta-tumor samples was defined as the Euclidean distance on all secretome concentrations, after natural logarithmic transformation. Groups were constructed from this continuous variable based on the distribution, using the quantiles 0.15 and 0.85.

#### Variables comparison and association

Comparison of a continuous variable among two groups was performed using a Student’s t test, paired when comparing tumor and juxta-tumor samples for the same patients, unpaired otherwise. Comparison between two categorical variables was performed using a* χ*2 test with Yates correction or a Fisher test, depending on the number of samples. Associations between variables were evaluated using Pearson correlation and were represented using the regression line from a linear regression model or using Spearman rank correlation in the presence of zero values before the preprocessing step. Only patients with values available for both tissues were displayed for representations displaying paired tumor and juxta-tumor samples.

#### Principal component analysis

For PCAs, missing data were imputed using a PCA model (missMDA R package [12]). PCA representations of the samples included group-specific 95% confidence ellipses based on a Gaussian distribution.

#### Secretome clustering

A clustering analysis was performed on the secretome analytes. Hierarchical clustering with Euclidean distance and Ward method was applied on the log-transformed ratios of concentration. A summary value was extracted for each patient, for each cluster of analytes: the mean of the log-transformed ratios.

#### ClueGO

Functional analysis was performed using Cytoscape’s ClueGO application (Version v2.5.5). Secretome analytes clusters identified in Fig. 3D were used as marker lists. Only the ImmuneSystemProcess-EBI-UniProt-GOA GOTerm ontology was used (updated on 08/01/2020), with the GO Term fusion option. Only pathways significant at the 0.05 level were kept. The minimum number of genes in the pathways was set to 2, and the minimum percentage of genes was set to 3%. The default value was used for all other options.

#### Tumor-specific signature

Preliminary tests were conducted using the secretome dataset to compare classification models. The elastic-net model was selected as the best compromise between performance on the data (cross-validation error), the possibility of interpreting the classifier, and computing time (compared with random forest, linear discriminant analysis, K-svm, kernel-KNN, XGBoost, bartMachine, using the SuperLearner wrapper). To build a tumor signature, the dataset was first randomly separated into train and test sets (70%-30% of the sample size). Paired tissues were attributed to the same set. On the train and test sets separately, near-zero variance variables were discarded, and multiple imputation was performed. Some clinical variables (surgery type, age at diagnosis, molecular class, number of invaded lymph nodes, presence of vascular emboli) and the outcome (tissue type) were included in the imputation model. To avoid automatic exclusion of variables, the predictor matrix was modified using mice::quickpred, with clinical features forced in the model. Thirty imputations were obtained with a maximum of 10 iterations, using predictive mean matching for continuous variables and logistic regression for categorical variables. Then, highly correlated variables were removed using the Caret methodology [13], with a correlation threshold of 0.8. Parameters of the elastic-net models were selected on the train set. The Lambda parameter was selected by tenfold cross-validation for each imputed dataset. The alpha parameter was selected based on AUC, accuracy, and numbers of parameters retained by the model. The final signature was obtained by keeping only variables selected in more than half of the imputed datasets. Specifically, the final estimate for a variable was the median of the estimates from the models that selected that variable. A classifier was obtained from the signature using the cutoff minimizing the distance between the ROC curve and point (0.1). Performances of the classifier were evaluated on the train set, as a control, and on the test set. Predicted probabilities, ROC curves, AUC, accuracy, sensitivity, specificity values, and confusion matrices were considered. The secretome-based classifier was also applied to a dataset of non-malignant tumors as a control. Associations of clinical features with the secretome-based signature were evaluated with ANOVA. Representations of the patients’ values were generated from the first imputed dataset.

### Software

Luminex data were analyzed using the Bio-Plex Manager 6.1 software. MSD data were analyzed using the Discovery workbench 4.0 software. Colorimetric assays were analyzed using the optima data analysis software version 3.32. Statistical analysis was performed using R software (version 3.5.3).

## Results

### Breast cancer patient cohort characteristics

**Fig. 1:**
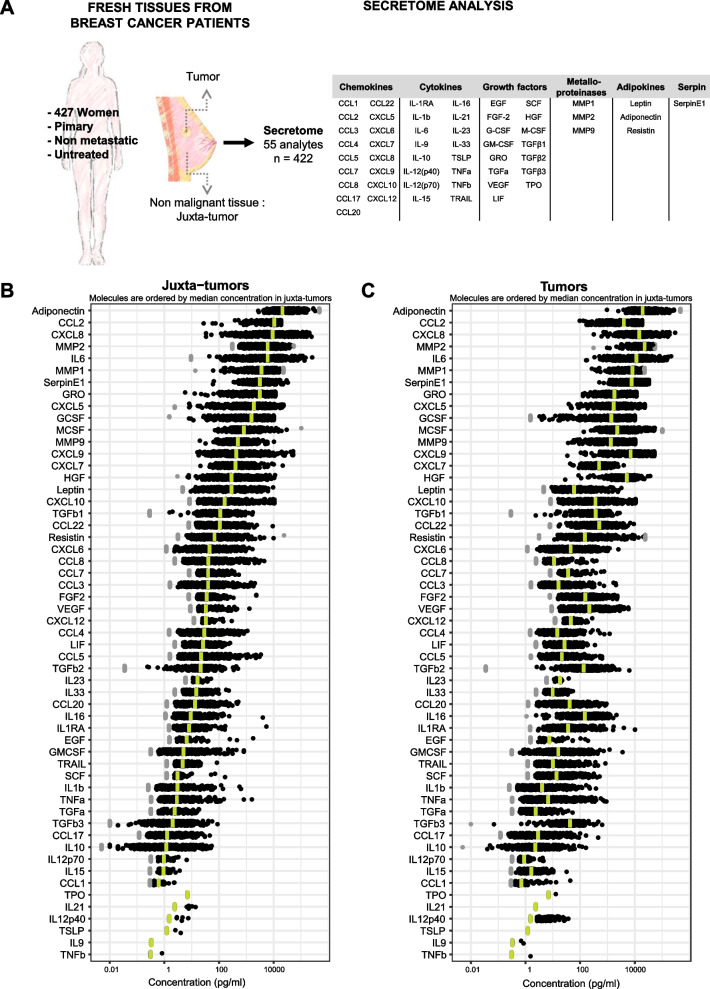
Secretome analysis of human breast tumor and juxta-tumor microenvironment **A** Schematic view of the experimental design and analysis. Primary breast cancer patients’ tumor tissues and paired non-malignant tissues (n = 422 patients, n = 844 samples) were freshly analyzed as explained in Methods section. Three datasets were generated: secretome analysis, glucose consumption and lactate production, and clinical data. The right panel shows the 55 analytes measured in this analysis. **B**, **C** Individual patient secretome concentration in pg/mL (n = 422 patients, except for molecules with technical issues for which samples with missing data were not displayed). All molecules were quantified by Luminex or MSD technologies in supernatants harvested after 24 h of tissue explant culture of juxta-tumor **B** and tumor tissues. **C** The molecules were ordered by median in-range concentration in the juxta-tumors. The green bars represent the median values of in-range concentrations. Samples outside of the detection range are displayed in gray. Molecules with less than 5% of in-range detection in juxta-tumors were represented in gray (IL-9, TPO, IL-12p40, TNF-β, TSLP, IL-21)

The tumor microenvironment global analysis (T-MEGA) was designed to deep profile tumor microenvironment (TME) inflammatory states of human breast cancer (BC). We analyzed 422 primary BC tumors and paired non-malignant breast tissues (hereafter referred to as juxta-tumors) from the same non-metastatic and untreated patient (Fig. 1A and Table 1). The inclusion criteria were as follows: woman, age > 18 years, a pathological diagnosis of BC, invasive tumors, absence of immune-modulating factor treatment (including steroids) within the past month, and absence of neoadjuvant therapy. Nearly half of the patients (46.8%, n = 200) were older than 60 years old at diagnosis, 37.5% (n = 160) were between 45 and 60 years old, and 15.7% (n = 67) were younger than 45 years old. Only 5.6% (n = 24) of the patients previously had another cancer (different location). However, 51.5% (n = 171) had family history of BC or ovarian cancer. Regarding the histologic type, 78% of the tumors were ductal carcinomas, and 12.2% were lobular carcinomas (Table 1). Tumors were grouped based on the molecular classification using hormone receptors (estrogen receptor, ER; progesterone receptor, PR), Her2 (human epidermal growth factor receptor 2), and Ki-67 expression. The cohort included 6.6% (n = 28) Her2 + (HER2, ER- PR- HER2 +), 30.8% (n = 130) luminal A (LUMA, ER + HER2- Ki-67 < 20%), 37.4% (n = 158) luminal B (LUMB, ER + HER2- Ki-67 ≥ 20%), 7.1% (n = 30) luminal Her2 + (LUMHER2, ER + HER2 +), and 18% (n = 76) triple negative BC (TN, ER- PR- HER2-) (Table 1 and S1). This was representative of the expected heterogeneity in BC molecular subtypes [9, 14, 15]. As previously documented, LUMA tumors are more often lobular carcinomas compared to the other molecular BC subtypes (25% versus less than 10%). Tumor pathological stages ranged from pT1a to pT4b, with a majority of pT1c and pT2 (42.2% and 49.6%, respectively). Half of the cohort presented pathological lymph node invasion. Last, 5.9% of the tumors were Elston–Ellis (EE) grade I, 43.5% grade II, and 50.6% grade III.

### Secretome profile distinguishes breast tumors from matched juxta-tumor tissues

**Fig. 2:**
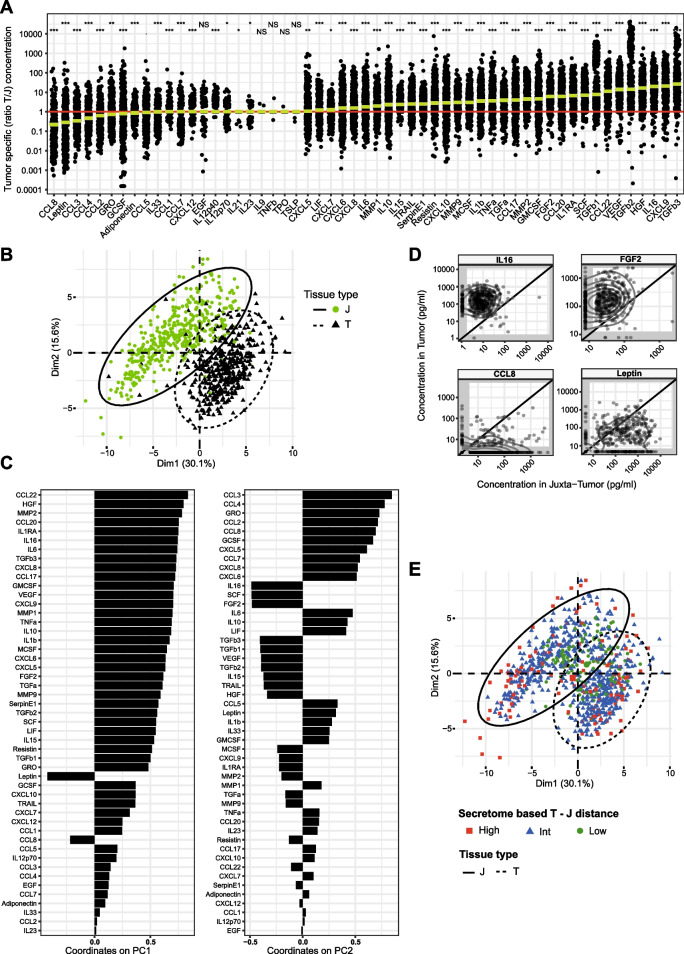
Secretome analysis allowed segregation between breast tumor and juxta-tumor tissues **A** Ratio between paired tumor and juxta-tumor molecules concentrations. The red line indicates the ratio of 1, meaning that the same analyte concentration was measured in the tumor and in the juxta-tumor supernatants. The molecules were ordered by the median value of the ratio (represented by the green bars). Molecules with less than 5% of in-range detection in juxta-tumors were represented in gray (IL-9, TPO, IL-12p40, TNF-β, TSPL, IL-21). Paired t test on the logged concentrations was performed, and p values were annotated as follow: NS (non-significant): > 0.05; *: ≤ 0.05; **: ≤ 0.01; ***: ≤ 0.001. (Detailed *P* values are reported in Table S4.) **B** Projection of the 422 tumors and 422 paired juxta-tumors on the first components of a PCA of the secretome data (after missing data imputation and without IL-9, TPO, IL-12p40, TNF-β, TSLP, IL-21). Confidence ellipses according to the tissue type were calculated from a Gaussian distribution at 95% level. **C** Bar plots describing the molecule contributions to the PCA components 1 and 2. **D** IL-16, FGF-2, CCL8, and Leptin concentrations measured in the juxta-tumor (X-axis) and the tumor tissue (Y-axis) supernatants for each patient. The two-dimensional density of the observations was displayed with iso-density contour lines. Regions outside of the detection range were displayed in gray. **E** Projection of the 422 tumors and 422 paired juxta-tumors on the first components of the same PCA as shown in B, with confidence ellipses according to the tissue type and color coded according to the three groups defined on the secretome-based distance between the tumors and the paired juxta-tumors

To analyze soluble BC TME characteristics, we performed an extensive secretome analysis of freshly resected tissue samples (Fig. 1A). Breast tumor and juxta-tumor supernatants were generated using a standardized protocol, as previously described in Ghirelli et al. [16]. Briefly, 15–20 mg of tissue was cultured in 250 µL of culture medium and supernatants were harvested 24 h later. First, we performed a quality control by quantifying glucose and lactate in supernatants of 38 matched tumor and juxta-tumor samples. As expected, higher glucose consumption and lactate production were observed in tumor supernatants as compared to the paired juxta-tumor samples (Fig. S1). The secretome profiling was performed by quantifying 16 cytokines, 17 chemokines, 15 growth factors, 3 metalloproteinases, 3 adipokines, and the serpin E1 (Fig. 1A, right panel), collectively covering key inflammatory processes and immune functions. Figure 1B and C displays the distribution of each secreted molecule in juxta-tumor and tumor tissues, respectively. Among the 55 analytes, IL-9, TPO, TNFβ, TSLP, IL-12p40, and IL-21 were undetectable in a large part of the samples (Fig. 1 and Table S2). In the remainder of the article, we focused our analysis on the 49 detected analytes and we asked whether a tumor-specific secretome landscape could be defined and would allow distinguishing tumor from non-malignant juxta-tumor tissues. A high heterogeneity in secretion levels across samples was observed (Table S3), and we decided to analyze the secretion ratio between tumor and paired juxta-tumor tissue, thereafter called tumor-specific, for each soluble analyte. This way, we could represent and interpret the tumor-specific change in secretion for each patient-derived sample (Fig. 2A). Among the 49 detected molecules, more than 70% were significantly more secreted by tumor as compared to juxta-tumor samples (Table S4). As previously described [16], GM-CSF, TNF-α, and IL-6 were detected at higher levels in the tumor supernatants as compared to the juxta-tumor ones. TGF-β [1–3], CXCL9, IL-16, HGF, and VEGF were the most differentially secreted. Among those, TGF-β1, 2, and 3 showed a bimodal distribution. This was due to detection limits for a proportion of juxta-tumor samples that were either secreting undetectable TGF-β levels or not secreting it at all (Fig. S2). CXCL5, CXCL6, CXCL7, CXCL8, and CXCL12 were slightly higher in tumors compared to juxta-tumors. The majority of the tumor supernatants (91.2%) had high CXCL9 levels, compared to their juxta-tumor counterparts. The metalloproteinase (MMP) 1, 2, and 9 and the cytotoxic molecule TRAIL were increased in tumor compared to juxta-tumor supernatants (Fig. 2A and Table S4). Only 20% of the analytes were significantly less secreted by the tumors compared to juxta-tumors, including Leptin, GRO, G-CSF, IL-33, and Adiponectin. Among CCL chemokines, CCL2, 3, 4, 7, and 8 were significantly less secreted by tumor tissues, while CCL17, CCL20, and CCL22 were secreted in higher concentrations in tumor as compared to juxta-tumor tissues. High heterogeneity between patients’ samples was observed for both tissue types, notably for Adiponectin, CXCL8, IL-6, MMP1, and MMP2 secretions. (Fig. 2A and Table S3).

A principal component analysis (PCA) revealed a strong separation between tumor and juxta-tumor samples (Fig. 2B). Tumors were separated from juxta-tumors partly on the first dimension of the PCA (30.1% of the variance), due notably to high secretions of CCL22, HGF, MMP2, CCL20, IL-1RA, and IL-16, and low secretions of Leptin and CCL8 (Fig. 2C). The second PCA dimension explained 15.6% of the variance and revealed a high heterogeneity in juxta-tumor samples. Overall, with this bidimensional analysis we could show that tumor samples were well characterized by high secretions of IL-16, HGF, the TGF-β1, 2 and 3, SCF, FGF-2 and VEGF, and low secretions of CCL8 and Leptin. The results of this multivariate analysis confirmed our findings on univariate analyses shown in Fig. 2A. IL-16, FGF-2, CCL8, and Leptin paired concentration were displayed, showing at the same time the secreted concentration for each patient and each tissue type (juxta-tumor on X-axis and tumor on Y-axis), and the distribution of the tumor-specific over- or under-expression (distance to the diagonal) (Fig. 2D). For almost all patients, IL-16 was detected at higher quantities in the tumor supernatant compared to the juxta-tumor, while the tumor-specific high FGF-2 levels were highly patient-dependent (Fig. 2D top panels). CCL8 and Leptin were specifically secreted in the juxta-tumor supernatants at heterogeneous concentrations (Fig. 2D, bottom panels). As tumors and juxta-tumors partially overlapped in the secretome-based PCA (Fig. 2B), we asked 1) if some tumor samples could display features similar to the juxta-tumor tissue and 2) if the samples at the intersection of the two groups systematically belonged to the same patients or were just a peculiar expression of inter-patient heterogeneity. To answer those questions, we computed a secretome-based multivariate distance between each tumor (T) and its paired juxta-tumor (J) sample based on the measurements of the 55 analytes. Three groups of T-J secretome-based distances were distinguished based on the distribution: high, intermediate, and low. “High” distance samples regrouped 15% of tumors with an overall secretome profile strongly different from their juxta-tumor. “Low” distance group consisted of 15% of tumors displaying a secretome similar to their juxta-tumor. The remaining 70% of paired samples belonged to the intermediate (“Int”) distance group (Fig. 2E). Low-distance samples were mostly found in the center of the graph, while high-distance samples were mostly found in the surroundings. However, at the intersection of the 2 PCA ellipses we could find all 3 types of samples. Overall, those samples did not systematically belong to the same patient. We could explain this behavior by describing two main profiles of tissue pairs at the intersection. The “low” distance pairs indicated a group of tumors that were not much differentiated. The “high” and “Int” distance samples formed a second group for which the given tissue type behaved like the other tissue type as a whole, without being similar to its paired counterpart. We referred to those samples as “Juxta-like tumors” or “Tumor-like juxta.”

Overall, those data revealed that the secretome efficiently distinguished tumors from matched juxta-tumor tissue.

### Tumor-specific secretome pattern

**Fig. 3:**
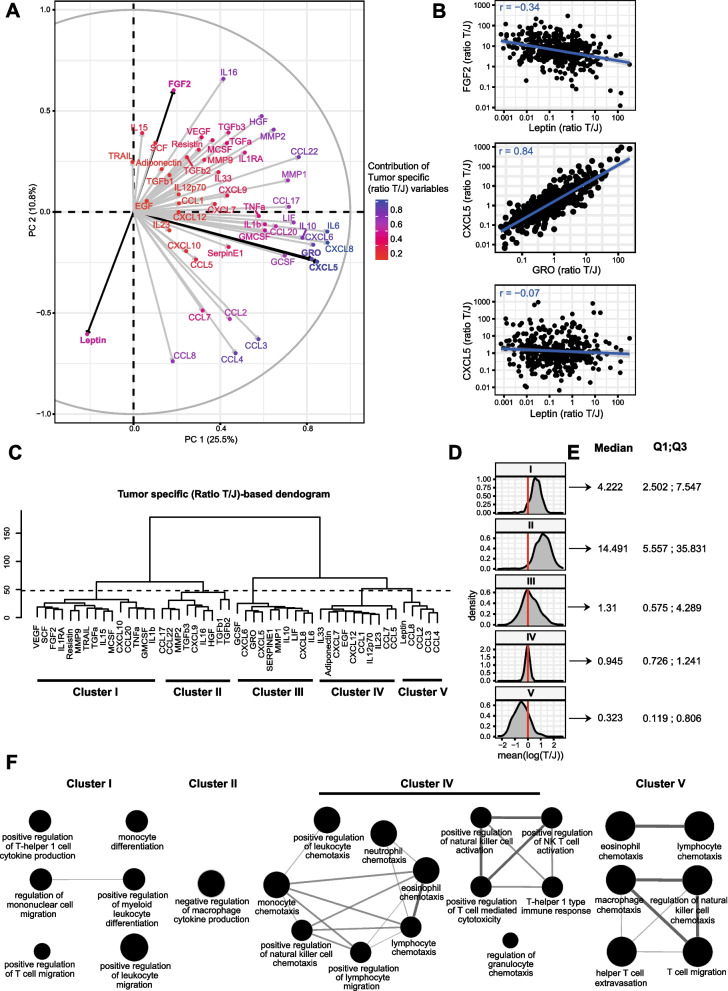
Breast tumor tissue-specific secretome pattern **A** Correlation circle from a PCA of tumor-specific secretome data. IL-9, TPO, IL-12p40, TNF-β, TSLP, and IL-21 were excluded from the analysis for insufficient detection. For each molecule, the coordinates of the arrow represent its contribution to each axis of the PCA. Global contribution levels (sum for PC1 and 2) were represented by a color scale. **B** Linear regression with 95% confidence interval with individual tumor-specific values (n = 422 patients) for three pairs of molecules: FGF-2/Leptin (negative association), CXCL5/GRO (positive association), CXCL5/Leptin (independent behavior). Pearson correlation coefficient of the logged tumor-specific values (*r*) is also displayed. **C** Dendrogram of the unsupervised hierarchical clustering of the secretome performed using the logged tumor-specific values of each sample. Five clusters were identified. **D** For each of those molecule clusters, the distribution of the 422 means tumor-specific logged values over the corresponding molecules was shown. **E** Median and quartile 1 and 3 values corresponding to the four metamolecules derived from the five clusters shown in panel **(C)**. **F** Immune pathways enriched at the 0.05 level, from a ClueGO analysis for each cluster. No pathway enrichment was found for cluster III. The node size is proportional to the significance of the enrichment; the edge size is proportional to Cohen’s Kappa score of interaction between two nodes

The tumor-specific secretion pattern was obtained by analyzing the tumor/juxta-tumor ratios of each analyte independently. Based on those data, we then performed a PCA to better understand the multivariate data structure of the tumor-specific secretome (Fig. 3A). The correlation circle from the PCA shows different types of associations: 1) strong positive associations, such as CXCL5 and GRO, 2) independent behaviors such as Leptin and CXCL5, and 3) a few negative associations, such as Leptin and FGF-2. (Fig. 3A and B).

Then, we performed an unsupervised clustering analysis of the concentration ratios to identify groups of molecules with similar tumor-specific secretion behavior. We identified five clusters on the dendrogram generated from a hierarchical clustering (Fig. 3C), from which we derived five metamolecules. Specifically, for each patient, the value of the metamolecule X was the mean of the concentration ratios of the molecules of the corresponding cluster X. We plotted the distribution of the five metamolecules and their associated values to quantify their secretory behaviors and to compare the clusters (Fig. 3D and E). Cluster I regrouped molecules secreted at higher levels by tumors than the paired juxta-tumors. Metamolecule I was in median four times higher in the tumor than the corresponding juxta-tumor. Cluster II regrouped molecules secreted at completely higher levels by tumors than the paired juxta-tumors: In median, metamolecule II was more than fourteen times higher in the tumors than the juxta-tumors. Metamolecules III, IV, and V were in median 1.31, 0.945, and 0.323 times higher in tumor than juxta-tumor, respectively. We then performed a ClueGO analysis to associate those five tumor-specific secretory behaviors to immune functional pathways (Fig. 3F). The associated functions for Cluster I were related to positive regulation of Th1 cells, monocyte differentiation, and migration of immune cells. Cluster II molecules were linked to immune suppression. This cluster was composed of molecules associated with negative macrophage regulation as well as Treg presence, notably CCL17 and CCL22, which are both involved in Treg recruitment through CCR4 engagement [17]. TGF-β family members, which are linked to immune suppression and tumor immune evasion [20, 21], were also part of this cluster. Cluster III regrouped molecules with, on average, the same secretion levels between tumors and paired juxta-tumors, but with a highly heterogeneous behavior across patients. No immune-related pathway was found enriched for that cluster. Cluster IV also regrouped molecules secreted in similar levels in tumors compared to paired juxta-tumors, but probably linked to shared functions, and corresponding to “Cytotoxicity and Chemotaxis” functions. Cluster V was composed of Leptin, CCL2, CCL3, CCL4, and CCL8, all being more secreted in the juxta-tumors as compared to paired tumors. This cluster corresponded to the following functions: eosinophil and macrophage chemotaxis, and regulation of immune cell migration or chemotaxis (Fig. 3F).

Overall, we identified clusters of analytes differing by their tumor-specific secretion and that were associated with immune-related functions.

### Tumor secretome defines a breast cancer signature

**Fig. 4:**
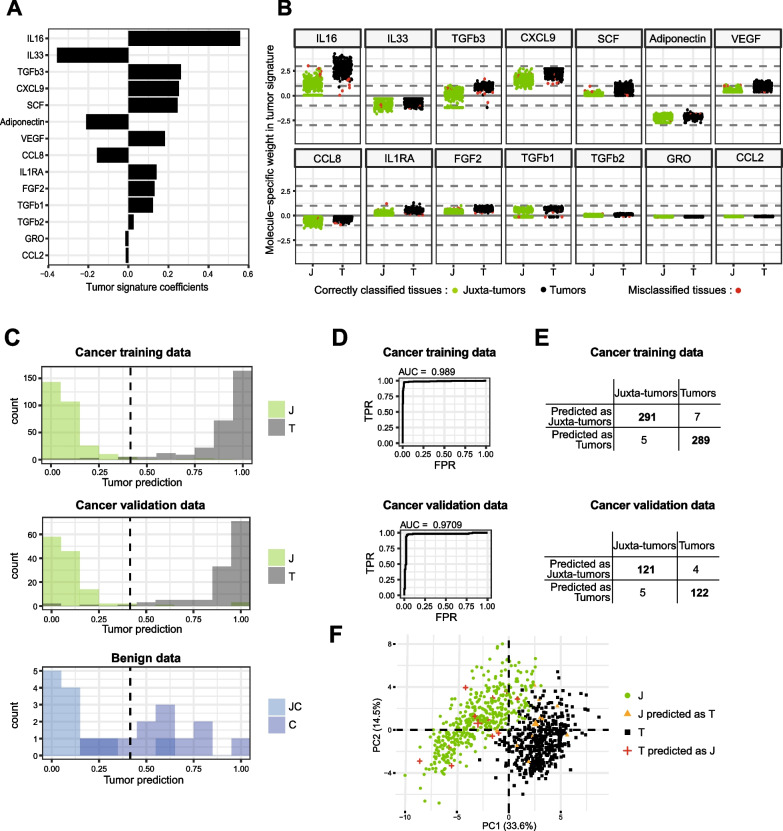
Immune secretome-based signature classifies breast tumor tissue from non-malignant juxta-tumor tissue **A** Bar plots describing the coefficients of the tumor signature. **B** With the train set: sample-specific values of the molecules weighted by the signature coefficients are displayed (n = 296 patients with paired tissues, first imputed dataset used). Samples that would be misclassified are displayed in red. **C** Histogram of the predicted probability of being a tumor for the samples of the train set (top panel, n = 296 patients with paired tissues), test set (middle panel, n = 126 patients with paired tissues), and for benign (C) and juxta-benign (JC) tissues (bottom panel, n = 12 patients with paired tissues). The cutoff value of the classifier is displayed as a dashed line. **D** ROC curves with AUC value evaluated on the train set (top panel) and test set (bottom panel). **E** Confusion matrix for the train set (top panel) and test set (bottom panel). **F** With the full dataset (train and test set): projection of the correctly predicted tumors (black) and juxta-tumors (green), misclassified tumors (orange) and juxta-tumors (red), on the two first axis of the secretome data PCA

We evaluated if the secretome could segregate breast tumors from non-malignant breast tissues. We performed a supervised learning analysis using an elastic-net modelization with a train/test strategy. The classifier was built on the train set, representing 70% of the secretome dataset (Fig. S3), and its performances were evaluated on the test set. Fourteen molecules were selected by the model: IL-16, IL-33, TGF-β3, CXCL9, SCF, Adiponectin, VEGF, CCL8, IL-1RA, FGF-2, TGF-β1, TGF-β2, GRO, and CCL2 (Fig. 4A). IL-33, Adiponectin, CCL8, GRO, and CCL2 contributed to the juxta-tumor type; the other analytes contributed to the tumor type. Altogether, this defined a tumor signature that can be interpreted as a “tumorness” score. Among the 14 molecules defining the signature, IL-16 was one of the most important in discriminating tumor versus juxta-tumor tissues, as shown by its high tumor signature coefficient (Fig. 4A). Figure 4B shows individual patient values for these molecules and highlights misclassification errors on the train set (red dots). Notably, misclassified tumors had low values on IL-16. Overall, high performances were obtained on the train set and on the test set. The histograms showed that most tissues were correctly predicted with very high confidence (Fig. 4C, top and middle panels). This was also illustrated by the ROC curve and AUC of 0.97 on the validation set (Fig. 4D), as well as accuracy, sensitivity, and specificity, which were all higher than 96% (Table 2). Indeed, only 4 tumors and 5 juxta-tumors out of 126 were misclassified on the test set (Fig. 4E). Moreover, when displaying all 422 patients on the 2 first components of a PCA, we observed that most misclassified samples were located in the cluster of the other tissue type, suggesting either outlier behavior or corrupted tissue labels (Fig. 4F). The tumor signature was also applied to 12 paired benign breast samples (cystic lesions). We observed that most benign juxta-cystic tissues were predicted as normal-like tissue. Most benign tumors were predicted as tumors but with low confidence, as evidenced by the shift of the distribution of tumor prediction between the malignant tumors and benign tumors (Fig. 4C, bottom panel). We further searched for associations of the continuous classifier with clinical features by performing ANOVAs (Table S5). Six features were found to have a significant association with the signature for tumor samples (Fig. S4). Notably, patients with at least one pregnancy previous to the cancer diagnosis had higher tumor signature values than patients with no pregnancy. Patients bearing tumors with lower risk factors (grade EE I vs. EE II and III, and low vs. high Ki-67) had tumors with lower signature values. Luminal A tumors had lower signature values than the other molecular subtypes.

**Table 2:** Classifiers’ performances on the train and test sets

	Secretome-based T-signature	
	Train set	Test set
T-signature threshold	≥ −0.344	
Tumor prediction threshold	≥ 0.415	
Accuracy	0.980	0.964
Sensitivity	0.976	0.968
Specificity	0.983	0.960

For each classifier cutoff on the signature scale and on the predicted probabilities used to define the classifier and related accuracy, sensitivity, and specificity values for the train and test sets

Overall, we defined a secretome-based signature that allows distinguishing tumor from juxta-tumor tissues with high sensitivity and specificity. IL-16, TGF-β3, CXCL9, and SCF were the best contributors to this tumor signature.

## Discussion

In this study, we provide for the first time a classifier to define breast tumor inflammation based on the multiparametric study of breast tissue-secreted molecules. Tissue microenvironment changes due to genetic alterations, epigenetic regulation, and inflammatory circuits are induced during the transition from a normal breast tissue to a cancerous tissue. Hence, the study of the juxta-tumor samples is of paramount importance: Understanding how a normal tissue behaves allows better characterizing and understanding specific tumor tissue components.

To perform the Tumor Microenvironment Global Analysis (T-MEGA), we optimized the sample management by ensuring tissue processing and culture within three hours from surgical resection. We decided to limit tissue manipulation in order to minimize cell death and avoid cellular stress and mechanical activation of inflammatory pathways. After interdisciplinary discussions, juxta-tumoral paired tissues were selected as the best control to assess specific tumor tissue components (same anatomical site, same patient). The tumor tissue delimitation has been defined macroscopically and microscopically by breast cancer specialized pathologists, and the juxta-tumor tissues included in our study were all tumor-free. However, juxta-tumor tissues used as non-involved breast tissue control do have limitations, including variations in tissue composition and variable distance to the tumor. In our study, no difference in the secretome profile was observed between juxta-tumors from tumorectomy compared to mastectomy. Furthermore, applying the tumor signature to the secretome profiles of 12 benign cystic breast lesions with their paired normal juxta-cystic breast counterparts showed that the juxta-cystic tissues presented a similar profile to the juxta-tumors ones, suggesting that juxta-tumor tissues were really closed to normal breast. Finally, the juxta-tumor secretome was not impacted by cancer molecular subtype.

Cytokines and other soluble mediators are often measured in serum samples of cancer patients [18, 19]. This represents a noninvasive approach, which allows collecting relatively large amounts of biological material and to perform multiplex measurements of soluble analytes. However, the quantification of circulating mediators has limitations because it does not reflect the changes occurring at the tissue microenvironment level. Breast tumor-protein studies have been mostly performed on the malignant tissue itself, without taking into account normal breast tissue composition, and without integrating data on immune-related molecules [20–23]. Several studies have compared tumor tissues and healthy counterparts relying exclusively on transcriptomic datasets and no information on secreted inflammatory proteins [9–11].

We set up the T-MEGA pipeline to characterize the soluble factors released by breast tissues. Multivariate analysis of breast tumor and juxta-tumor secretome allowed us to compute a tumor-specific signature that led to a clear discrimination of the malignant tissue from its normal counterpart. Those findings were strengthened by the validation of our model on a small cohort of benign tumor samples, such as cystic lesion samples.

Among the key features forming the breast cancer classifier, we found molecules widely known to be involved in tumor immune evasion, such as TGF-b family members. Even though they are classically associated with an immune suppression phenotype, a dual role in inhibiting and promoting tumors has been extensively described for different TGF-b family members [24, 25]. Other molecules are currently less studied in the context of breast tissue. This is the case for IL-16, which is known to play a role in regulating T cell growth, activation and motility [26]. Few literature reports associated the role of IL-16 with the progression of different cancer types, one of those being breast cancer [26], but the presence of IL-16 at the breast cancer microenvironment tissue level has never been shown so far. Our analysis not only showed for the first time the presence of IL-16 in breast tumor tissue, but also demonstrated the specificity of IL-16 to the breast tumor microenvironment relative to the non-malignant counterpart.

By deeply analyzing the secretome of breast tumor tissue in comparison with its non-involved counterpart, we identified novel breast tissue features and achieved three important goals: 1) the characterization of the non-involved breast tissue soluble microenvironment; 2) the formalization of a breast cancer-specific classifier, based on clinically applicable and quantifiable secretome features; and 3) the generation of a freely accessible resource to help the biomedical community in advancing scientific knowledge on breast cancer. Our dataset could further be used to help answering other biomedical questions, in particular according to histopathological and molecular subtypes.

Overall, our study opens new horizons for personalized anticancer therapy design by providing a reference on primary untreated breast cancer secretome. Differences in secretome profile could be used for diagnostic, prognostic, or as predictive biomarker. Changes in secretome profile can also be used for drug assessment [27, 28] or could allow identification of new therapeutic targets in treatment resistant patients. The T-MEGA framework also opens broad perspectives for the study of other cancer types and should help better delineating biological features that are specific of the tumoral process.

## Conclusions

Our study represents a comprehensive and systematic evaluation of the breast cancer tissue and the normal breast tissue. We have used a systematic experimental approach on a large (> 400) breast tumor patient cohort (T-MEGA) to generate a unique secretome dataset. In order to precisely define tissue “tumorness,” we established a novel and widely applicable breast cancer-specific classifier by mathematically formalizing. Our dataset is a freely accessible resource to the biomedical community to help advancing scientific knowledge on breast cancer. Moreover, the key elements of the signature that we have identified could open new approaches to the design of targeted strategies in the context of breast cancer. The pipeline that we have built is applicable to other cancer type studies as well as diverse inflammatory diseases.

## Supplementary Information

**Additional file 1**: **Fig S1**. Glucose and lactate dosage in supernatants. Paired tumor and juxta-tumor ratios of glucose and lactate concentrations (μmol/mg) measured in supernatants after 24h of culture (n=38 patients). Each dot represents a paired measurement. The green bars represent the median; the red line highlights a ratio of 1, meaning that the two breast tissue types display the same amount of glucose consumption or lactate production.

**Additional file 2**: **Fig S2**. TGF-β bimodal distribution. TGF-β1, 2 and 3 concentrations measured in the juxta-tumor (X-axis) and the tumor tissue (Y-axis) supernatants for each patient. The 2-dimensional density of the observations was displayed with iso-density contour lines. Regions outside of the detection range were displayed in gray.

**Additional file 3**: **Fig S3**. Train and test sets represented on a secretome PCA. Train set, shown in orange, represented the 70% of the secretome dataset, and was used to build the breast tumor classifier. Validation set is shown in light blue.

**Additional file 4**: **Fig S4** Clinical features significantly associated to the tumor signature value. Associations between the signature value of tumor samples and six clinical features selected by ANOVA test. Median and interquartile range are displayed. P-values were annotated as follow: •: ≤0.1; *: ≤0.05; **: ≤0.01; ***: ≤0.001. Detailed p-values associated to all clinical features analyzed are shown in Table S5.

**Additional file 5**: **Table S1** Patients' characteristics according to breast cancer molecular subtypes. **Table S2** Luminex and MSD technical thresholds. **Table S3** Variability of quantified secretome molecules in breast cancer tumor and juxta-tumor supernatants. **Table S4** Secretome comparison of breast tumor and paired juxta-tumor samples. **Table S5** Association of clinical parameters and the tumor secretome-based signature.

**Additional file 6**: **Table S6** Databases: the dataset with imputation of data according to limit of detection (LOD) as explained in material and methods, the raw dataset (with ND for values below detection limit), the table S2 and a summary of used abbreviations in the dataset.

## Data Availability

The data generated or analyzed during this study are available in Table S6. The dataset supporting our conclusions is included within the article as a supplementary excel table (Table S6). It contains several sheets: the dataset with imputation of data according to limit of detection (LOD) as explained in material and methods, the raw dataset (with ND for values below detection limit), Table S2, and a summary of used abbreviations in the dataset.

## Abbreviations

T-MEGA: Tumor microenvironment global analysis
TGF-β1: Transforming growth factor β1
TGF-β2: Transforming growth factor β2
TGF-β3: Transforming growth factor β3
RNAseq: Ribonucleic acid sequencing
BC: Breast cancer
CO_2_: Carbon dioxide
RPMI: Roswell Park Memorial Institute medium
FBS: Fetal bovine serum
MMP: Matrix Metalloproteinase
CCL1: Chemokine (C-C motif) ligand 1
CCL2: Chemokine (C-C motif) ligand 2
CCL3: Chemokine (C-C motif) ligand 3
CCL4: Chemokine (C-C motif) ligand 4
CCL5: Chemokine (C-C motif) ligand 5
CCL7: Chemokine (C-C motif) ligand 7
CCL8: Chemokine (C-C motif) ligand 8
CCL17: Chemokine (C-C motif) ligand 17
CCL20: Chemokine (C-C motif) ligand 20
CCL22: Chemokine (C-C motif) ligand 22
CXCL5: Chemokine (C-X-C motif) ligand 5
CXCL6: Chemokine (C-X-C motif) ligand 6
CXCL7: Chemokine (C-X-C motif) ligand 7
CXCL8: Chemokine (C-X-C motif) ligand 8
CXCL9: Chemokine (C-X-C motif) ligand 9
CXCL10: Chemokine (C-X-C motif) ligand 10
CXCL12(a + b): Chemokine (C-X-C motif) ligand 12(a + b)
EGF: Epidermal growth factor
FGF-2: Fibroblast growth factor 2
GCSF: Granulocyte colony-stimulating factor
GM-CSF: Granulocyte-macrophage colony-stimulating factor
HGF: Hepatocyte growth factor
IL-1β: Interleukin 1 beta
IL-1RA: Interleukin-1 receptor antagonist
IL-6: Interleukin 6
IL-9: Interleukin 9
IL-12p40: Interleukin 12 subunit p40
IL-12p70: Interleukin 12 subunit p70
IL-15: Interleukin 15
IL-16: Interleukin 16
IL-21: Interleukin 21
IL-23: Interleukin 23
IL-33: Interleukin 33
LIF: Leukemia inhibitory factor
M-CSF: Macrophage colony-stimulating factor
MMP-1: Matrix metalloproteinase-1
MMP-2: Matrix metalloproteinase-2
MMP-9: Matrix metalloproteinase-9
SCF: Stem cell factor
TGF-α: Transforming growth factor alpha
TNF-α: Tumor necrosis factor alpha
TNF-β: Tumor necrosis factor beta
TPO: Thyroid peroxidase
TSLP: Thymic stromal lymphopoietin
VEGF: Vascular endothelial growth factor
IL-10: Interleukin 10
MSD: Mesoscale discovery
PCA: Principal component analysis
GO: Gene Ontology
ROC: Receiver operating characteristic
AUC: Area under the curve
TME: Tumor microenvironment
ER: Estrogen receptor
PR: Progesterone receptor
HER2: Human epidermal growth factor receptor 2
LUMA: Luminal A
LUMB: Luminal B
TN: Triple negative
EE: Elston–Ellis
CCR4: Chemokine (C-C motif) receptor 4
ANOVA: Analysis of variance
