# Supplementary figures and images for: Definition of a novel breast tumor-specific classifier based on secretome analysis
Source: Breast Cancer Res. 2022 Dec 20;24:94. doi: 10.1186/s13058-022-01590-4 (PMC9764559; doi:10.1186/s13058-022-01590-4)

# Ratio Tumor/Juxta-Tumor

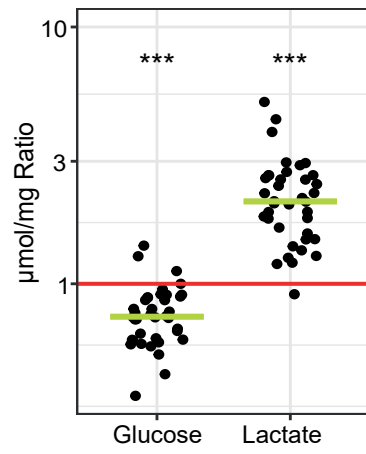

Supplement: Supplementary file 1 — Additional file 1: Fig S1. Glucose and lactate dosage in supernatants. Paired tumor and juxta-tumor ratios of glucose and lactate concentrations (μmol/mg) measured in supernatants after 24h of culture (n=38 patients). Each dot represents a paired measurement. The green bars represent the median; the red line highlights a ratio of 1, meaning that the two breast tissue types display the same amount of glucose consumption or lactate production. [file 13058_2022_1590_MOESM1_ESM.pdf]

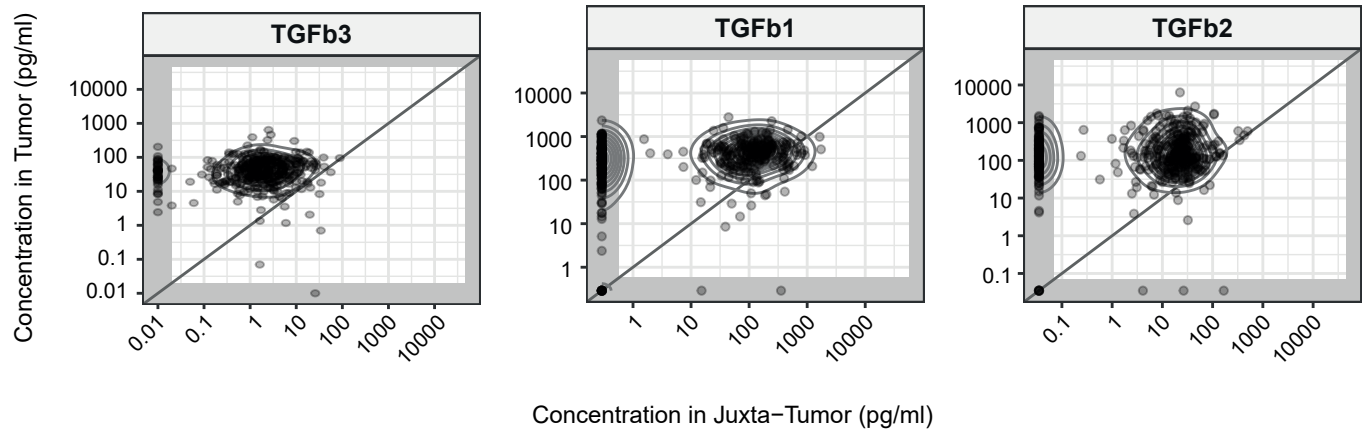

Supplement: Supplementary file 2 — Additional file 2: Fig S2. TGF-β bimodal distribution. TGF-β1, 2 and 3 concentrations measured in the juxta-tumor (X-axis) and the tumor tissue (Y-axis) supernatants for each patient. The 2-dimensional density of the observations was displayed with iso-density contour lines. Regions outside of the detection range were displayed in gray. [file 13058_2022_1590_MOESM2_ESM.pdf]

Secretome based tumor signature

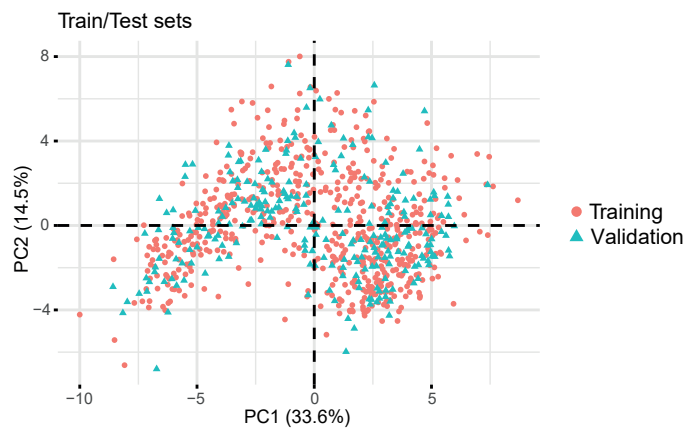

Supplement: Supplementary file 3 — Additional file 3: Fig S3. Train and test sets represented on a secretome PCA. Train set, shown in orange, represented the 70% of the secretome dataset, and was used to build the breast tumor classifier. Validation set is shown in light blue. [file 13058_2022_1590_MOESM3_ESM.pdf]

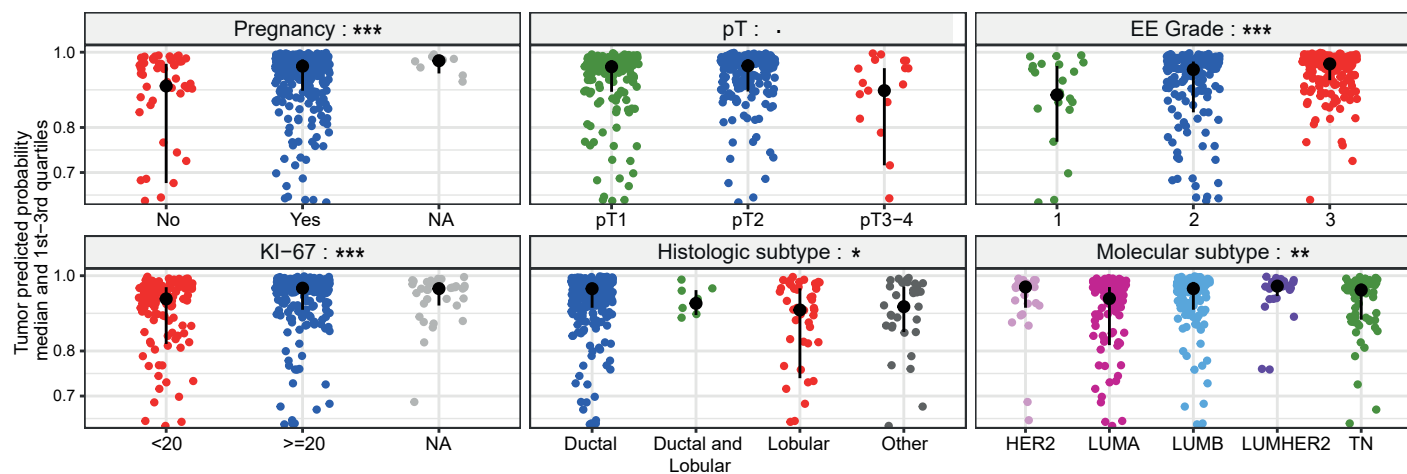

Supplement: Supplementary file 4 — Additional file 4: Fig S4 Clinical features significantly associated to the tumor signature value. Associations between the signature value of tumor samples and six clinical features selected by ANOVA test. Median and interquartile range are displayed. P-values were annotated as follow: •: ≤0.1; *: ≤0.05; **: ≤0.01; ***: ≤0.001. Detailed p-values associated to all clinical features analyzed are shown in Table S5. [file 13058_2022_1590_MOESM4_ESM.pdf]
