# Supplementary material for: Definition of a novel breast tumor-specific classifier based on secretome analysis
Source: Breast Cancer Res. 2022 Dec 20;24:94. doi: 10.1186/s13058-022-01590-4 (PMC9764559; doi:10.1186/s13058-022-01590-4)
Supplement: Supplementary file 5 — Additional file 5: Table S1 Patients' characteristics according to breast cancer molecular subtypes. Table S2 Luminex and MSD technical thresholds. Table S3 Variability of quantified secretome molecules in breast cancer tumor and juxta-tumor supernatants. Table S4 Secretome comparison of breast tumor and paired juxta-tumor samples. Table S5 Association of clinical parameters and the tumor secretome-based signature. [file 13058_2022_1590_MOESM5_ESM.docx]

**SUPPLEMENTARY TABLES S1, S2, S3, S4 and S5**

**Table S1.** Patients' characteristics according to breast cancer molecular subtypes.

| Breast Cancer molecular classification | | | | | | | | |
| --- | --- | --- | --- | --- | --- | --- | --- | --- |
|  |  | **HER2** | **LUMA** | **LUMB** | **LUMHER2** | **TN** | P-value | |
| Age at diagnosis | <45 | 6 (21%) | 22 (17%) | 20 (13%) | 9 (30%) | 10 (13%) | 0.049 | * |
|  | 45-60 | 12 (43%) | 46 (35%) | 70 (44%) | 11 (37%) | 21 (27%) |  |  |
|  | ≥60 | 10 (36%) | 65 (49%) | 69 (43%) | 10 (33%) | 46 (60% |  |  |
| BMI | Underweight | 1 (4%) | 6 (5%) | 9 (6%) | 2 (7%) | 6 (8%) | 0.836 |  |
|  | Healthy weight | 15 (56%) | 63 (47%) | 77 (51%) | 13 (43%) | 36 (49%) |  |  |
|  | Overweight | 7 (26%) | 38 (29%) | 42 (28%) | 9 (30%) | 25 (34%) |  |  |
|  | Obesity | 4 (15%) | 26 (20%) | 23 (15%) | 6 (20%) | 6 (8%) |  |  |
|  | NA | 1 | 0 | 8 | 0 | 4 |  |  |
| Pregnancy | No | 2 (7%) | 23 (18%) | 26 (17%) | 4 (13%) | 6 (8%) | 0.233 |  |
|  | Yes | 25 (93%) | 107 (82%) | 127 (83%) | 26 (87%) | 70 (92%) |  |  |
|  | NA | 1 | 3 | 6 | 0 | 1 |  |  |
| Menopause | No | 6 (26%) | 38 (32%) | 35 (26%) | 15 (52%) | 17 (24%) | 0.053 | . |
|  | Yes | 17 (74%) | 81 (68%) | 101 (74%) | 14 (48%) | 54 (76%) |  |  |
|  | NA | 5 | 14 | 23 | 1 | 6 |  |  |
| HRT (Hormone Replacement Therapy) | No | 7 (64%) | 34 (64%) | 48 (62%) | 6 (55%) | 19 (58%) | 0.958 |  |
|  | Yes | 4 (36%) | 19 (36%) | 29 (38%) | 5 (45%) | 14 (42%) |  |  |
|  | NA | 17 | 80 | 82 | 19 | 44 |  |  |
| Personal history (K) | No | 28 (100%) | 126 (95%) | 151 (95%) | 29 (97%) | 69 (90%) | 0.317 |  |
|  | Yes | 0 (0%) | 7 (5%) | 8 (5%) | 1 (3%) | 8 (10%) |  |  |
| Family history (BC, OVK) | No | 11 (50%) | 47 (44%) | 64 (55%) | 13 (54%) | 26 (42%) | 0.338 |  |
|  | Yes | 11 (50%) | 61 (56%) | 52 (45%) | 11 (46%) | 36 (58%) |  |  |
|  | NA | 6 | 25 | 43 | 6 | 15 |  |  |
| pT | pT1 | 15 (54%) | 63 (47%) | 72 (45%) | 12 (40%) | 31 (40%) | 0.803 |  |
|  | pT2 | 12 (43%) | 60 (45%) | 80 (50%) | 17 (57%) | 43 (56%) |  |  |
|  | pT3-4 | 1 (4%) | 10 (8%) | 7 (4%) | 1 (3%) | 3 (4%) |  |  |
| pN | pN0 | 16 (57%) | 64 (48%) | 74 (47%) | 9 (30%) | 47 (61%) | 0.041 | * |
|  | pN1 | 11 (39%) | 47 (35%) | 59 (37%) | 10 (33%) | 22 (29%) |  |  |
|  | pN2 | 0 (0%) | 13 (10%) | 20 (13%) | 7 (23%) | 4 (5%) |  |  |
|  | pN3 | 1 (4%) | 9 (7%) | 6 (4%) | 4 (13%) | 4 (5%) |  |  |
| EE grade | 1 | 0 (0%) | 21 (16%) | 2 (1%) | 0 (0%) | 2 (3%) | <0.001 | *** |
|  | 2 | 5 (18%) | 93 (71%) | 70 (44%) | 8 (27%) | 9 (12%) |  |  |
|  | 3 | 23 (82%) | 17 (13%) | 87 (55%) | 22 (73%) | 66 (86%) |  |  |
|  | NA | 0 | 2 | 0 | 0 | 0 |  |  |
| Ki-67 (%) | <20 | 1 (5%) | 133 (100%) | 0 (0%) | 3 (15%) | 6 (11%) | <0.001 | *** |
|  | >=20 | 20 (95%) | 0 (0%) | 159 (100%) | 17 (85%) | 51 (89%) |  |  |
|  | NA | 7 | 0 | 0 | 10 | 20 |  |  |
| Histological subtype | Ductal | 25 (89%) | 88 (66%) | 130 (82%) | 27 (90%) | 63 (82%) | <0.001 | *** |
|  | Other | 2 (7%) | 12 (9%) | 14 (9%) | 2 (7%) | 12 (16%) |  |  |
|  | Lobular | 1 (4%) | 33 (25%) | 15 (9%) | 1 (3%) | 2 (3%) |  |  |
| Vascular emboli | No | 14 (50%) | 86 (66%) | 80 (50%) | 13 (43%) | 48 (62%) | 0.031 | * |
|  | Yes | 14 (50%) | 45 (34%) | 79 (50%) | 17 (57%) | 29 (38%) |  |  |
|  | NA | 0 | 2 | 0 | 0 | 0 |  |  |

Number of samples and percentage of patients per level of the categorical clinical variables for each molecular subtype. Chi-square with Yates correction or Fisher test was performed, depending on the sample size.

LUMA: luminal A; LUMB: luminal B HER2-; LUMHER2: HR+ HER2+; HER2: HR-HER2+; TN: triple negative (HR-HER2-); BMI: body mass index.

**Table S2.** Luminex and MSD technical thresholds.

|  | **Lower detection limit** | **Higher detection limit** |  |  | **Lower detection limit** | **Higher detection limit** |
| --- | --- | --- | --- | --- | --- | --- |
| **IL-1b** | 0.45 | 8594.20 |  | **TGFβ2** | 0.07 | 45400.00 |
| **CCL1** | 0.36 | 2095.60 |  | **CCL8** | 4.60 | 5064.03 |
| **IL-15** | 0.45 | 10115.09 |  | **CCL5** | 2.86 | 10108.96 |
| **TNFβ** | 0.49 | 10028.11 |  | **FGF2** | 12.77 | 2317.46 |
| **IL-12p70** | 0.58 | 10173.03 |  | **CCL4** | 2.38 | 10996.70 |
| **TGFa** | 0.59 | 2130.63 |  | **CXCL6** | 2.15 | 2516.46 |
| **IL-9** | 0.62 | 10443.64 |  | **CCL3** | 2.56 | 2049.37 |
| **CCL17** | 0.22 | 1022.23 |  | **CCL22** | 14.32 | 10988.14 |
| **IL-10** | 0.01 | 327.00 |  | **TGFβ1** | 0.58 | 56600.00 |
| **SCF** | 2.30 | 10462.38 |  | **Resistin** | 5.71 | 23679.25 |
| **TSLP** | 2.40 | 12728.50 |  | **CXCL10** | 15.82 | 10680.35 |
| **IL-12p40** | 2.31 | 10301.15 |  | **Leptin** | 6.88 | 35135.77 |
| **EGF** | 2.25 | 10550.22 |  | **HGF** | 5.56 | 100939.93 |
| **TGFβ3** | 0.02 | 45800.00 |  | **CXCL7** | 18.59 | 21493.87 |
| **TNFa** | 0.54 | 10564.02 |  | **CXCL9** | 11.19 | 51651.20 |
| **IL-21** | 3.53 | 24918.61 |  | **MMP9** | 12.68 | 10278.54 |
| **TRAIL** | 2.28 | 10184.00 |  | **MCSF** | 21.92 | 100944.52 |
| **GMCSF** | 0.56 | 10444.58 |  | **CXCL5** | 3.77 | 23072.31 |
| **IL-1RA** | 2.26 | 10217.39 |  | **GCSF** | 2.28 | 10000.53 |
| **CCL20** | 2.31 | 10375.94 |  | **SerpinE1** | 8.14 | 33027.46 |
| **IL-23** | 9.52 | 50178.83 |  | **GRO** | 2.49 | 11433.61 |
| **IL-33** | 4.68 | 23503.74 |  | **MMP2** | 556.39 | 51608.86 |
| **TPO** | 11.02 | 53973.94 |  | **IL-6** | 14.70 | 360420.30 |
| **IL-16** | 1.74 | 11732.22 |  | **MMP1** | 26.59 | 20758.10 |
| **CCL7** | 15.01 | 10771.30 |  | **CXCL8** | 18.90 | 300814.20 |
| **VEGF** | 15.71 | 10272.90 |  | **CCL2** | 4.45 | 18678.47 |
| **LIF** | 4.44 | 20991.54 |  | **Adiponectin** | 18.23 | 440489.33 |
| **CXCL12** | 22.63 | 107103.02 |  |  |  |  |

Molecules in black were quantified using Luminex technology; molecules in blue were quantified using MSD technology**Table S3.** Variability of quantified secretome molecules in breast cancer tumor and juxta-tumor supernatants.

|  | **Standard deviation for Juxta-tumors** | **Standard deviation for Tumors** |  |  | **Standard deviation for Juxta-tumors** | **Standard deviation for Tumors** |
| --- | --- | --- | --- | --- | --- | --- |
| **IL-9** | 0 | 0.031 |  | **FGF2** | 1.6e+02 | 3.9e+02 |
| **TPO** | 0 | 0.29 |  | **CXCL6** | 1.9e+02 | 2.1e+02 |
| **TNFb** | 0.025 | 0.059 |  | **TGFb1** | 1.9e+02 | 3.4e+02 |
| **TSLP** | 0.15 | 0 |  | **IL-16** | 2e+02 | 2.6e+02 |
| **CCL1** | 0.19 | 2.3 |  | **IL-1RA** | 2.6e+02 | 7.4e+02 |
| **IL-15** | 0.32 | 2.3 |  | **CCL3** | 2.7e+02 | 1.7e+02 |
| **IL-12p40** | 0.37 | 4.1 |  | **CCL5** | 3.1e+02 | 69 |
| **IL-12p70** | 0.47 | 0.47 |  | **CCL8** | 4e+02 | 35 |
| **IL-21** | 0.81 | 0 |  | **CCL22** | 5.3e+02 | 1e+03 |
| **TGFa** | 2.6 | 4.4 |  | **MMP9** | 8.9e+02 | 3e+03 |
| **CCL17** | 4.5 | 26 |  | **Leptin** | 1.1e+03 | 3.3e+02 |
| **IL-23** | 4.7 | 2.8 |  | **Resistin** | 1.4e+03 | 3e+03 |
| **TRAIL** | 5.3 | 60 |  | **HGF** | 1.6e+03 | 3.8e+03 |
| **IL-10** | 7.6 | 13 |  | **CXCL7** | 1.6e+03 | 4.3e+02 |
| **TGFb3** | 7.6 | 55 |  | **CXCL10** | 1.9e+03 | 2.4e+03 |
| **SCF** | 9.1 | 55 |  | **CXCL5** | 3.5e+03 | 4.2e+03 |
| **CXCL12** | 20 | 27 |  | **GCSF** | 3.8e+03 | 3.5e+03 |
| **IL-33** | 21 | 8.1 |  | **GRO** | 3.9e+03 | 3.7e+03 |
| **LIF** | 36 | 38 |  | **SerpinE1** | 4.1e+03 | 8.5e+03 |
| **IL-1b** | 43 | 30 |  | **CCL2** | 5.3e+03 | 6e+03 |
| **TGFb2** | 45 | 4.6e+02 |  | **MCSF** | 5.5e+03 | 1.4e+04 |
| **CCL20** | 55 | 1.3e+02 |  | **CXCL9** | 7.6e+03 | 1.9e+04 |
| **CCL7** | 57 | 57 |  | **MMP1** | 8.2e+03 | 8e+03 |
| **GMCSF** | 70 | 2e+02 |  | **MMP2** | 8.8e+03 | 1.4e+04 |
| **VEGF** | 81 | 7.3e+02 |  | **IL-6** | 2.4e+04 | 2.7e+04 |
| **TNFa** | 88 | 86 |  | **CXCL8** | 3.6e+04 | 3.6e+04 |
| **EGF** | 90 | 14 |  | **Adiponectin** | 8.8e+04 | 5.9e+04 |
| **CCL4** | 1.3e+02 | 80 |  |  |  |  |

*.*

**Table S4.** Secretome comparison of breast tumor and paired juxta-tumor samples.

|  | **Estimate*** | **P-value*¤** | |  |  | **Estimate*** | **P-value*¤** |  |
| --- | --- | --- | --- | --- | --- | --- | --- | --- |
| **CCL8** | -0.7191 | 9.1e-48 | *** |  | **MMP1** | 0.3635 | 2.71e-26 | *** |
| **Leptin** | -0.6537 | 3.71e-33 | *** |  | **IL10** | 0.3959 | 2.59e-17 | *** |
| **CCL3** | -0.4379 | 1.09e-32 | *** |  | **IL15** | 0.4105 | 1.39e-59 | *** |
| **CCL4** | -0.3197 | 7.66e-21 | *** |  | **TRAIL** | 0.4396 | 1.45e-38 | *** |
| **CCL2** | -0.3510 | 4.33e-23 | *** |  | **SerpinE1** | 0.4015 | 1.33e-56 | *** |
| **GRO** | -0.0984 | 0.00422 | ** |  | **Resistin** | 0.5029 | 7.36e-27 | *** |
| **GCSF** | -0.2079 | 0.000232 | *** |  | **CXCL10** | 0.4052 | 1.88e-14 | *** |
| **Adiponectin** | -0.0618 | 0.00036 | *** |  | **MMP9** | 0.4948 | 1.06e-46 | *** |
| **CCL5** | -0.0622 | 0.0885 | . |  | **MCSF** | 0.5193 | 2.81e-60 | *** |
| **IL33** | -0.2109 | 2.08e-16 | *** |  | **IL1b** | 0.4954 | 8.41e-30 | *** |
| **CCL1** | 0.0638 | 3.5e-06 | *** |  | **TNFa** | 0.5719 | 3.67e-41 | *** |
| **CCL7** | -0.1456 | 2.97e-08 | *** |  | **TGFa** | 0.4783 | 4.72e-45 | *** |
| **CXCL12** | 0.0878 | 7.29e-08 | *** |  | **CCL17** | 0.6128 | 3.14e-37 | *** |
| **EGF** | 0.0246 | 0.196 | NS |  | **MMP2** | 0.7248 | 1.25e-74 | *** |
| **IL12p40** | 0.1461 | 1.15e-21 | *** |  | **GMCSF** | 0.6036 | 3.77e-35 | *** |
| **IL12p70** | 0.0246 | 0.0438 | * |  | **FGF2** | 0.7877 | 7.06e-100 | *** |
| **IL21** | -0.0073 | 0.0281 | * |  | **CCL20** | 0.7120 | 1.65e-53 | *** |
| **IL23** | -0.0157 | 0.0356 | * |  | **IL1RA** | 0.8629 | 1.02e-104 | *** |
| **IL9** | 0.0018 | 0.161 | NS |  | **SCF** | 0.8537 | 8.86e-92 | *** |
| **TNFb** | 0.0006 | 0.739 | NS |  | **TGFb1** | 1.3860 | 2.44e-70 | *** |
| **TPO** | 0.0006 | 0.318 | NS |  | **CCL22** | 0.9927 | 9.66e-81 | *** |
| **TSLP** | -0.0021 | 0.169 | NS |  | **VEGF** | 1.1034 | 5.65e-108 | *** |
| **CXCL5** | 0.1199 | 0.00174 | ** |  | **TGFb2** | 1.5630 | 4.81e-77 | *** |
| **LIF** | 0.1993 | 8.61e-08 | *** |  | **HGF** | 1.1429 | 2.17e-117 | *** |
| **CXCL7** | 0.0710 | 0.0108 | * |  | **IL16** | 1.2648 | 1.2e-133 | *** |
| **CXCL6** | 0.1767 | 9.79e-05 | *** |  | **CXCL9** | 1.2352 | 9.08e-104 | *** |
| **CXCL8** | 0.2738 | 3.68e-13 | *** |  | **TGFb3** | 1.4858 | 3.5e-113 | *** |
| **IL6** | 0.3420 | 3.63e-15 | *** |  |  |  |  |  |

** Estimate and P-value of paired student test on the logged concentration comparing tumor and juxta-tumor samples. Positive estimates indicate larger concentrations in the tumors.*

*¤ P-values were annotated as follow: NS (non significative): >0.05; *: ≤0.05; **: ≤0.01; ***: ≤0.001.*

**Table S5.** Association of clinical parameters and the tumor secretome-based signature.

| **Features** | **Levels** | **n** | **Mean (sd)** | **P-value** |  |
| --- | --- | --- | --- | --- | --- |
| Age at diagnosis | <45 | 65 | 0.9 (0.17) | 0.59 |  |
|  | 45-60 | 159 | 0.88 (0.19) |  |  |
|  | ≥60 | 198 | 0.89 (0.16) |  |  |
| BMI | Underweight | 23 | 0.91 (0.12) | 0.13 |  |
|  | Healthy weight | 201 | 0.86 (0.19) |  |  |
|  | Overweight | 121 | 0.91 (0.15) |  |  |
|  | Obesity | 64 | 0.9 (0.18) |  |  |
| Pregnancy | No | 61 | 0.8 (0.23) | <0.0001 | *** |
|  | Yes | 351 | 0.9 (0.16) |  |  |
| Menopause | No | 109 | 0.9 (0.17) | 0.35 |  |
|  | Yes | 264 | 0.88 (0.18) |  |  |
| HRT (Hormone Replacement Therapy) | No | 114 | 0.88 (0.19) | 0.66 |  |
|  | Yes | 71 | 0.87 (0.19) |  |  |
| Personal history (K) | No | 399 | 0.88 (0.18) | 0.60 |  |
|  | Yes | 23 | 0.9 (0.11) |  |  |
| Family history (BC, OVK) | No | 159 | 0.9 (0.16) | 0.15 |  |
|  | Yes | 169 | 0.87 (0.19) |  |  |
| Relapse event | No | 373 | 0.89 (0.17) | 0.92 |  |
|  | Yes | 49 | 0.88 (0.19) |  |  |
| pT | pT1 | 193 | 0.89 (0.18) | 0.055 | . |
|  | pT2 | 208 | 0.89 (0.16) |  |  |
|  | pT3-4 | 21 | 0.8 (0.23) |  |  |
| pN | pN0 | 208 | 0.89 (0.18) | 0.93 |  |
|  | pN1 | 146 | 0.88 (0.17) |  |  |
|  | pN2-3 | 68 | 0.89 (0.16) |  |  |
| EE grade | EE I | 25 | 0.83 (0.17) | 0.0008 | *** |
|  | EE II | 182 | 0.86 (0.18) |  |  |
|  | EE III | 213 | 0.91 (0.15) |  |  |
| Ki-67 | <20% | 140 | 0.84 (0.2) | 0.0007 | *** |
|  | ≥20% | 245 | 0.91 (0.15) |  |  |
| Histologic subtype | Ductal | 331 | 0.9 (0.17) | 0.030 | * |
|  | Ductal and Lobular | 8 | 0.87 (0.18) |  |  |
|  | Lobular | 50 | 0.82 (0.2) |  |  |
|  | Other | 33 | 0.86 (0.2) |  |  |
| Vascular emboli | No | 236 | 0.89 (0.16) | 0.93 |  |
|  | Yes | 184 | 0.88 (0.19) |  |  |
| Molecular subtype | HER2 | 28 | 0.91 (0.12) | 0.009 | ** |
|  | LUMA | 130 | 0.84 (0.21) |  |  |
|  | LUMB | 158 | 0.9 (0.16) |  |  |
|  | LUMHER2 | 30 | 0.93 (0.14) |  |  |
|  | TN | 76 | 0.9 (0.15) |  |  |
| BRCA1/2 mutation | No | 38 | 0.85 (0.25) | 0.82 |  |
|  | Yes | 10 | 0.87 (0.11) |  |  |
